# Transforaminal Blood Patch for the Treatment of Chronic Headache from Intracranial Hypotension: A Case Report and Review

**DOI:** 10.1155/2012/923904

**Published:** 2011-07-18

**Authors:** Kirk Bowden, Adam Wuollet, Amol Patwardhan, Theodore J. Price, John Lawall, Jeffery Annabi, Steven Barker, Emil Annabi

**Affiliations:** ^1^Department of Anesthesiology and Pain Management, University of Arizona, 1501 N. Campbell Avenue, Room 5301, P.O. Box 245114, Tucson, AZ 85724, USA; ^2^Department of Pharmacology, University of Arizona, Tucson, AZ 85724, USA; ^3^Department of Neurology, University of Arizona, Tucson, AZ 85724, USA; ^4^El Paso Orthopaedic Surgery Group, El Paso, TX 79930, USA

## Abstract

This case report describes the successful treatment of chronic headache from intracranial hypotension with bilateral transforaminal (TF) lumbar epidural blood patches (EBPs). The patient is a 65-year-old male with chronic postural headaches. He had not had a headache-free day in more than 13 years. Conservative treatment and several interlaminar epidural blood patches were previously unsuccessful. A transforaminal EBP was performed under fluoroscopic guidance. Resolution of the headache occurred within 5 minutes of the procedure. After three months without a headache the patient had a return of the postural headache. A second transforaminal EBP was performed again with almost immediate resolution. The patient remains headache-free almost six months from the time of first TF blood patch. This is the first published report of the use of transforaminal epidural blood patches for the successful treatment of a headache lasting longer than 3 months.

## 1. Introduction

**Figure 1:**
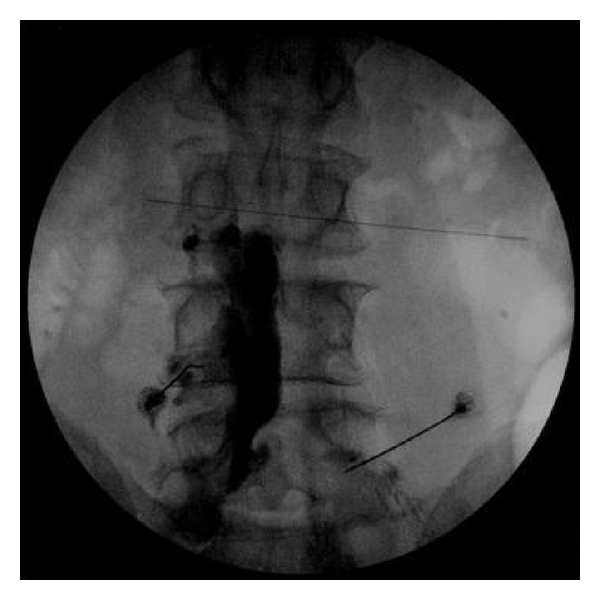
Fluoroscopic image of epidural contrast injected through right L4-L5 foramen.

**Figure 2:**
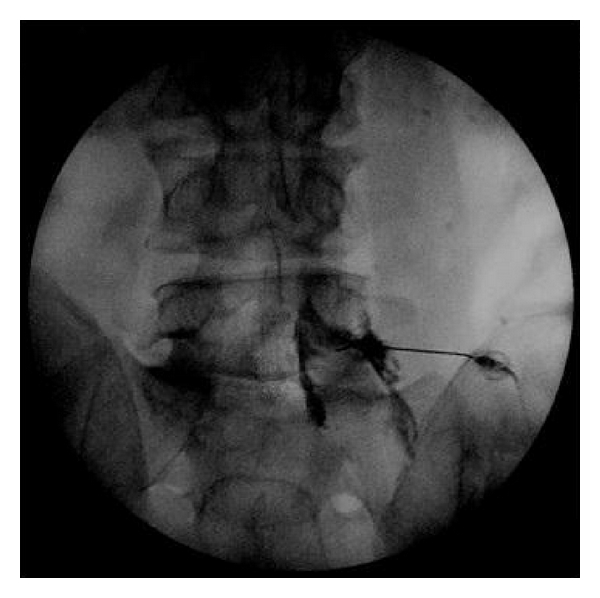
Fluoroscopic image of epidural contrast injected through left L5-S1 foramen.

Headaches secondary to intracranial hypotension or cerebrospinal fluid hypovolemia have been well documented for over 100 years. Dr. Bier experienced such a headache first hand in 1898 which lead to the first report of what is now known as postdural puncture headache (PDPH) [1, 2]. Forty years later Dr. Schaltenbrand described spontaneous intracranial hypotension (SIH) [3] which has recently become a more recognized cause of severe persistent headache. PDPH and SIH are very similar in mechanism, symptomatology as well as treatment. A relative decrease in intracranial pressure is thought to cause irritation of pain sensitive structures such as the meninges and bridging veins. Patients typically present with a postural occipital-frontal headache that resolves in the supine position and is greatly exacerbated by sitting or standing. The headaches can be associated with neck pain, nausea, vomiting photophobia, and cranial nerve palsies [4–6]. In severe cases, SIH has been associated with dementia, encephalopathy, paralysis, coma, and even death [7–9]. In 2004 the International Classification of Headache Disorders, 2nd edition provided specific diagnostic criteria for SIH [10]. These criteria are shown in Table 1. Conservative therapy including bed rest, oral hydration, increased salt intake along with intravenous fluid, caffeine, and the use of an abdominal binder have all been recommended [4, 6]. Refractory cases of both PDPH and SIH typically resolve with the use of an epidural blood patch (EBP). Dr. Gormley described this technique in 1960 and it remains the treatment of choice when conservative management has been ineffective [4, 6, 11]. Traditionally, EBP is performed by placing a needle in the epidural space through an interlaminar approach and injecting 10–30 mL of sterile autologous blood. At times the traditional interlaminar approach is either impractical due to surgical scar or local infection. We present a case of successful treatment of chronic headache secondary to SIH using a transforaminal epidural blood patch (Figures 1 and 2). Using a transforaminal approach allowed for placement of blood directly at the presumed site of CSF leak when an interlaminar approach was not practical because of a previous laminectomy. 

## 2. Case Report

This patient is a 65-year-old male with a history of chronic postural headache for 13 years. The headaches started after sustaining a ground level fall in 1997 shortly after having a L4-L5 laminectomy in 1997 for spinal stenosis. He was eventually seen by a specialist in low pressure headaches and was subsequently diagnosed with spontaneous intracranial hypotension. Computed tomographic melography (CTM) demonstrated a likely CSF leak at L4-L5. The headaches were initially managed conservatively with bed rest, caffeine, increase oral intake, intravenous fluid, and an abdominal binder. These measures provided only minimal temporary relief. Multiple interlaminar epidural blood patches were performed but none of them were effective. The patient also underwent C6–C8 rhizotomy as well as multiple C2-C3 epidural steroid injections. Discouraged and not wanting to consider surgical intervention the patient decided to simply try and cope with the pain. He continued to use acetaminophen, ibuprofen, and oxycodone 40 mg q12 hrs but continued to have daily headaches. Unable to tolerate the headaches any longer the patient once again sought medical intervention in 2010. At that time he was referred to the current authors for evaluation and potential nonsurgical intervention. 

At the time of consultation the patient complained of daily dull, achy frontal headache with some radiation to the neck that was significant worse when sitting or standing and resolved when lying supine. His pain was reported to be 9/10 with verbal numeric rating scale (VNRS). The headaches are frequently associated with recent nausea, vomiting, and photophobia. On physical exam he was found to be afebrile, normotensive, and with no gross neurological deficits. Heavily T2-weighted magnetic resonance myelography (MRM) was performed which showed a CSF collection in the posterior epidural space at the level of L5 presumably representing the site of CSF leak. MRM was chosen to help located the exact site of CSF leak because addition lumbar puncture for intrathecal contrast for a CTM could exacerbate the patient's symptoms [12]. The case was discussed with the patient's neurosurgeon and the decision was made to attempt an EBP by entering the bilateral intervertebral foramen. The potential risks and benefits were explained to the patient in great detail. 

## 3. Procedure Note

After written consent was obtained the patient was brought to the operating room and placed in the prone position. The skin was prepped and draped in the usual sterile fashion and a skin wheal was raised with 3 mL of 1% lidocaine. Under real-time fluoroscopic guidance the L5 pedicle was identified. A 25 gauge spinal needle was inserted but could not be advanced into the L5-S1 intervertebral foramen. After two attempts, the needle was withdrawn and inserted at the level of L4. The needle was then advanced to the 6 o'clock position of the L4 pedicle. Contrast was then injected and epidural spread was identified. 15 mL of sterile autologous blood was then injected into the epidural space. The injection was stopped as the patient began to feel pressure in his lower back but no pain or paraesthesias were reported. On the left side a 25 gauge spinal needle was easily inserted in the 6 o'clock position of the L5 pedicle. After injection of contrast 15 mL of sterile autologous blood was injected. A total of 30 mL of sterile autologous blood was injected. After remaining prone for approximately 5 minutes the patient was moved to the seated then standing position. For the first time in 13 years the patient was able to stand without a headache. 

## 4. Patient Followup

The patient was seen at two weeks and two months for followup and found to be completely headache-free with no apparent complications from the procedure. Three months after the procedure the patient began having slight headaches when he would stand. The headaches were much less severe than before the procedure. They were described as 5/10 on VNRS with frontal “pressure.” He denied radiation of pain, nausea, vomiting, and photo- or phonophobia. Treatment options were discussed with the patient and the decision was made to repeat the transforaminal EBP. The procedure was repeated using the exact same technique. 15 mL of sterile autologous blood was injected through the intervertebral foramen at L4 on the right and then an additional 15 mL at L5 on the left. Again, within 5 minutes of the procedure the patient was completely headache-free in both the seated and standing positions. The patient was contacted by phone two months after the second epidural blood patch at which time he reported no return of symptoms. 

## 5. Discussion

An extensive literature review produced only 3 published reports of successful treatment of intracranial hypotension or PDPH using transforaminal epidural blood patch in addition to the current paper [9, 13, 14]. A transforaminal approach was also used by Schievink et al. who reported 4 cases of injection of a fibrin sealant into the epidural space for the treatment of SIH. Two of the 4 patients had a resolution of symptoms one of which had headaches for 8 months [15]. To our knowledge this is the first reported case of successful treatment chronic headache using transforaminal EBP. Each of the published cases is summarized in the Table 1 including patient characteristics, preprocedure diagnosis, duration of symptoms, site, the use of contrast, and the quantity of autologous blood injected. The current case was included in Table 2 for comparison. Of note, no complications were reported in any of the cases. The most common complication of EBP is low back pain. Other reported potential complications of EBP include aseptic meningitis, radicular pain, lumbovertebral syndrome, bradycardia, fever, subdural hematoma, epidural hematoma, and seizures [16]. 

Two of the transforaminal EBPs were performed for PDPH following transforaminal epidural steroid injection (ESI). The other case was for the treatment of refractory SIH. While each of the cases reported resolution of headache there was a wide range of the quantity of autologous blood injected into the epidural space. Weil et al. had a resolution of symptoms after only 8 total ml of blood injected while the current authors used 30 mL [13]. In 3 of the 4 cases interlaminar EBP had been attempted at least twice. The reason for successful treatment of both SIH and PDPH using a transforaminal approach when previous interlaminar EBP had failed is not exactly clear. We believe this is likely a function of the ability to place blood in close proximity to the dural defect.

A transforaminal approach for the EBP was chosen for the current case to obtain a more direct approach to the dural leak. We felt a direct interlaminar approach at L4-L5 would be unsafe as the integrity of the ligamentum flavum was likely compromised during the laminectomy. The lack of an intact ligamentum flavum would increase the possibility of inadvertent dural puncture and potential worsening of symptoms. An interlaminar approach at a level above or below the defect would likely be ineffective as this had previously been attempted. The two prior EBPs at L2-L3 and through a caudal approach, respectively, were likely ineffective because they failed to reach the site of CSF leak. The spread of epidural blood was likely limited because of postsurgical adhesions. Entering the intervertebral foramen allowed us to avoid possible adhesions and place blood directly at the site of the CSF leak. 

Headaches related to intracranial hypotension either from dural puncture or SIH can be severe and very difficult to treat. EBP appears to be the treatment of choice when conservative measures has failed. When EBP does not provide relief patient may benefit from surgical intervention if the site of the CSF leak has been identified [17]. In the case presented the patient suffered from a chronic postural headache for more than 13 years despite medical management and repeated interlaminar EBP. He was referred to clinic as he did not want to consider surgery. The use of a relatively novel approach to a treatment that has been used for 50 years eliminated the patient's headache and restored his quality of life. 

## 6. Conclusion

This case demonstrates that transforaminal epidural blood patch can be an effective in the treatment of chronic headache secondary to intracranial hypotension when traditional interlaminar technique is either impractical or has been previously ineffective. 

## Figures and Tables

**Table 1:** Diagnostic criteria for headache due to spontaneous spinal CSF leak and intracranial hypotension according to the International Classification of Headache Disorders, 2nd edition, 2004 [10].

(A) Diffuse and/or dull headache that worsens within 15 min after sitting or standing, with at least one of the following and fulfilling criterion D:	

(1) Neckstiffness	
(2) Tinnitus	
(3) Hypacusia	
(4) Photophobia	
(5) Nausea	

(B) At least one of the following:	

(1) Evidence of low CSF pressure on MRI (e.g., pachymeningeal enhancement)	
(2) Evidence of CSF leakage on conventional myelography, CT myelography or cisternography	
(3) CSF opening pressure <60 mm H_2_O in sitting position	

(C) No history of dural puncture or other cause of CSF fistula	

(D) Headache resolves within 72 h after epidural blood patching	

**Table 2:** Summary of published case reports of transforaminal EBP.

Author	Age	Sex	Preprocedure diagnosis	Duration of symptoms	Site	Contrast	Quantity of blood injected	Result	Previous interlaminar EBP
Weil	48	M	PDPH s/p Transforaminal ESI	5 weeks	Left L4-L5 L5-S1	No	2 mL each level	Relief within 5 min	Interlaminar EBP not attempted
Slipman	40	F	PDPH s/p Transforaminal ESI	3 months	Left C5-C6	Yes	6 mL	Relief within 15 min	Previous failed Interlaminar EBP ×2
Walega	39	F	SIH	8 weeks	Bilateral C7-T1	Yes	5 mL Left 2 mL Right	Relief time not reported	Previous failed Interlaminar EBP ×2
Bowden	65	M	SIH	13 years	Bilateral L4-L5	Yes	15 mL Bilateral ×2	Relief within 5 min	Multiple previous Interlaminar EBP
